# Gravity’s effect on biology

**DOI:** 10.3389/fphys.2023.1199175

**Published:** 2023-07-03

**Authors:** S. Anand Narayanan

**Affiliations:** Department of Nutrition and Integrative Physiology, Florida State University, Tallahassee, FL, United States

**Keywords:** gravity, space biology, space life sciences, gravitational biology, plant biology, animal biology

## Abstract

Gravity is a fundamental interaction that permeates throughout our Universe. On Earth, gravity gives weight to physical objects, and has been a constant presence throughout terrestrial biological evolution. Thus, gravity has shaped all biological functions, some examples include the growth of plants (e.g., gravitropism), the structure and morphology of biological parts in multicellular organisms, to its effects on our physiological function when humans travel into space. Moreover, from an evolutionary perspective, gravity has been a constant force on biology, and life, to our understanding, should have no reason to not experience the effects of gravity. Interestingly, there appear to be specific biological mechanisms that activate in the absence of gravity, with the space environment the only location to study the effects of a lack of gravity on biological systems. Thus, in this perspective piece, biological adaptations from the cellular to the whole organism levels to the presence and absence of gravity will be organized and described, as well as outlining future areas of research for gravitational biological investigations to address. Up to now, we have observed and shown how gravity effects biology at different levels, with a few examples including genetic (e.g., cell cycle, metabolism, signal transduction associated pathways, etc.), biochemically (e.g., cytoskeleton, NADPH oxidase, Yes-associated protein, etc.), and functionally (e.g., astronauts experiencing musculoskeletal and cardiovascular deconditioning, immune dysfunction, etc., when traveling into space). Based from these observations, there appear to be gravity-sensitive and specific pathways across biological organisms, though knowledge gaps of the effects of gravity on biology remain, such as similarities and differences across species, reproduction, development, and evolutionary adaptations, sex-differences, etc. Thus, here an overview of the literature is provided for context of gravitational biology research to-date and consideration for future studies, as we prepare for long-term occupation of low-Earth Orbit and cis-Lunar space, and missions to the Moon and Mars, experiencing the effects of Lunar and Martian gravity on biology, respectively, through our Artemis program.

## 1 Introduction

Gravity has been a constant presence throughout the history of Earth, and while invisible to the human eye, has provided pivotal evolutionary direction for life on Earth. Gravity is a vector, a force that has magnitude and direction in space, with gravitational loading on Earth being directed towards the Earth’s center. Gravity effects every object on Earth and defines the weight of each object based from the object’s mass. Weight is the mass times the force of gravity, with weight being variable for an object depending on the object’s position with respect to the gravity vector. Moreover, biological objects are living organisms with unique morphologies, physiology, behavior, and geographic locations, with their biological processes adaptable over time and in response to their environment, including to the gravitational vector. These adaptations also have varying magnitudes and directions, based on the organisms’ intrinsic and extrinsic properties. Indeed, with life on Earth being exposed to gravity, gravity has and continues to have significant effects on biological structure, function, and evolution.

Moreover, when considering the evolution of life, we must consider water as a requirement for life on Earth, and take note of life originating from bodies of water (Hug et al., 2016; do Nascimento Vieira et al., 2020). In aquatic environments, neutral buoyancy balances organisms’ weight against the force of gravity. While aquatic organisms can sense gravity, when organisms moved to the terrestrial surface and began to live on land for the first time, these organisms became gravitationally loaded without neutral buoyancy supporting their weight provided by an aquatic environment (Watanabe et al., 1991). In turn, the terrestrial environment led to adaptations of biological mechanisms unique to the specific effects of gravity. Moreover, as organisms increased in size, mass, and moved to different locations (e.g., altitudes), gravity continued to be influential, leading to new structures and functions of cells, tissues, organs, and organisms in synergy with environmental effects. Now when biology travels into space, significant changes occur from experiencing a lack of gravity and adapting to the unique space environment. Here a brief overview from selected relevant literature of these adaptations are described, and put forth as considerations for the effects of and lack of gravity on biological structure and function, as well as outlining future suggested areas of research to further delineate gravity’s distinct role on biology.

## 2 Gravity’s effects on life

Biology’s ability to adapt and evolve when exposed to gravity is related to the organisms size, mass, and position with respect to the gravity vector. For example, single cells and microbes can withstand 10^5 G’s, plants 30–40 G’s, rats 15G’s, and humans 4-5G’s, highlighting organisms’ increasing sensitivity to gravity as the organism increases in size and mass (Morey-Holton, 2003).

### 2.1 Cellular adaptations to gravity

Gravity’s effects on cells are varied, being location dependent (e.g., intracellular *versus* extracellular), and unique to the cell type (e.g., animal *versus* plant cells). Cells themselves are made up of different components, including the nucleus, organelles (e.g., mitochondria, Golgi body, endoplasmic reticulum, etc.), cytoskeleton, cell membrane, etc., all of which have mass and are in turn reactive to the gravity vector. Moreover, a cell’s function is homeostatically regulated based on its structure, composition, and orientation of its different parts. One example is the cytoskeleton, which is involved with maintaining cellular shape and serving as a scaffold for all cellular parts. Indeed, there exist cellular biochemical pathways sensitive to gravity (e.g., mechanosensitive pathways), which vary depending on the cell type (e.g., bone, muscle, heart, skin, etc.). One example of an animal cell mechanosensitive pathway includes the transcriptional coactivator Yes-associated protein (YAP). YAP’s relationship with gravity was discovered from analysis of the medaka fish YAP mutant; the mutant body was observed to be flattened because of its inability to adapt to gravity (Dupont et al., 2011; Porazinski et al., 2015). Additional pathways involved with YAP include cytoskeletal (e.g., F-actin) and cell growth and proliferation (e.g., Hippo) (Sudol et al., 1995; Kanai et al., 2000; Dong et al., 2007; Yu and Guan, 2013). Indeed, it has been observed F-actin polymerization activates YAP and its target gene, ARHGAP18. ARHGAP18 in turn negatively regulates F-actin polymerization, which then suppresses YAP activity. This is a negative feedback loop, with negative regulation of F-actin polymerization by YAP optimizing F-actin turnover and maximizing actomyosin contractility, which regulates 3D cellular structure and orientation (Hirata et al., 2015). Thus, YAP, as one example pathway described here, along with other proteins (e.g., focal adhesion proteins, p130Cas, Talin, etc.) serve as gravity sensitive proteins. In short, animal cells in an environment without gravity show decreased F-actin and YAP expression, effecting their cellular morphometry (Vorselen et al., 2014).

### 2.2 Genetic adaptations to gravity

Regulating cellular machinery (e.g., proteins) is gene expression, and various studies have observed genes sensitive to gravity changes. Examples include cytoskeletal gene expression pathways altered from spaceflight exposure such as calponin, dynactin, tropomodulin, keratin 8, myosin, RhoGTPases, ankyrin EST, plectin, and C-NAP1 (Lewis et al., 2001). One study showed 91 genes (e.g., NF-κB, CREB, ELK, AP-1, STAT, etc.) inhibited after simulated microgravity exposure (Ward et al., 2006). Another study showed 89 genes changed their expression due to simulated microgravity exposure, including genes involved with signal transduction, apoptosis, structure-transport-binding protein genes, tissue growth regulation, metabolic pathway regulation, as well as histone, nucleotide binding, RNA-binding protein and DNA repair genes (Sundaresan and Pellis, 2009). Cellular growth pathways also shift due to a lack of gravity; one study showed the cell cycle control protein p21 to be affected by microgravity exposure, while another study observed inhibition of the Rel/NF-KB, CREB, REL, and SRF pathways, which regulate cell cycle progression (Chang et al., 2012). Cellular metabolism adaptations have also been seen in response to a lack of gravity, with changes in reactive oxygen species, antioxidant factors, and cellular response to oxidative stress conditions (Tauber et al., 2018). An example of a direct microgravity effect on cellular structure and function includes a study that showed microgravity effecting NADPH oxidase, a membrane-bound multiprotein complex closely associated with cytoskeletal dynamics and oxidative phosphorylation (Dubinin and Vaulina, 1976; Nauseef et al., 1991; El Benna et al., 1994; Louis et al., 2015; Thiel et al., 2017). In short, there are diverse effects resulting from a lack of gravity on cell biology at different biochemical levels, including gene expression, protein functional, intracellular communication, cellular structural, etc. However, knowledge gaps exist across these domains, and more research would help elucidate gravity’s comprehensive and holistic role with biochemical structure and function, as well as how these processes evolved given the energy demands of being weighted *versus* weightless.

### 2.3 Tissue adaptations to gravity

Cellular adaptations to gravity determine tissue adaptations to gravity, as well. Specifically with mammals, bone is one example, having cells (e.g., osteocytes) that sense and adapt to different states of gravitational loading. Indeed, osteocytes maintain bone homeostasis by balancing bone resorption and formation processes in response to different states of mechanical loading. Osteocytes are mechanically stimulated by shear stresses across their cell bodies, and subsequently produce different secretory factors effecting cells (e.g., osteoblasts, osteoclasts) responsible for bone resorption and formation (Metzger and Narayanan, 2019). With a simulated microgravity model, osteocytes exposed to different mechanical loading conditions were observed to activate mechano-transduction biochemical pathways including ion channels, connexins, integrins, and cytoskeletal molecules. One specific example includes the protein p130Cas, an osteoclast mechano-sensing molecule involved with regulation of bone homeostasis, which translocates to the nucleus with decreased loading (i.e., weightlessness resulting from a lack of gravity) and negatively regulates NF-KB activity to suppress bone resorption (Sawada et al., 2006; Wittkowske et al., 2016; Yavropoulou and Yovos, 2016; Miyazaki et al., 2019). Muscle is another tissue mechano-sensitive to gravity, with muscle wasting occurring in spaceflight due to a lack of gravity. Studies of muscle myoblasts experiencing microgravity have shown activation and reduction of TRPC channels (e.g., TRPC1, TRPC3, and TRPC6), which in turn have been described as gravity sensitive proteins (Benavides Damm et al., 2013; Numaga-Tomita et al., 2019).

Indeed, from experiments flown into space, ground experiments experiencing simulated spaceflight conditions, and astronauts traveling into space, we have been able to observe how an absence of gravity induces numerous cellular, molecular, biochemical, and physiological adaptations. A lack of gravity effects the entire organism and all of its compartments, and when people travel to space, causing significant physiological implications to their health (reviewed in greater detail in reference Stepanek et al., 2019). For example, cardiovascular adaptations to reduced gravitational exposure includes decreased blood volume, reduction in blood pressure, reduced left-ventricular volume, orthostatic intolerance, and reduced cardiac contractility (Buckey et al., 1985; Patel, 2020). Orthostatic intolerance occurs frequently with crewmembers, with many astronauts experiencing the side-effects of orthostatic intolerance such as syncope and tachycardia. Another medical condition resulting from a lack of gravity due to spaceflight exposure includes spaceflight associated neuro-ocular syndrome, which is characterized by optic disc edema, choroidal and retinal folds, flattening of the posterior region of the sclera, and hyperopic refractive error shifts; in short, vision loss (Lee et al., 2020). Immune dysfunction also occurs from spaceflight exposure, such as reduced T and NK cell function, changes with monocytes and granulocytes, and fluctuations in cytokine levels, leading to a chronic inflammation phenotype with astronauts (Crucian et al., 2014; Crucian et al., 2016; Mehta et al., 2017; Ponomarev et al., 2017). Experiments have shown mechanical forces stimulate immune cells such as T cells, highlighting again potential gravity-sensitive mechanism with these cells as well (Hauschild et al., 2014).

### 2.4 Invertebrate adaptations to gravity

Having already described bones, unique to vertebrates, invertebrates, lacking an internal skeleton, are also animals responsive to gravity. While most invertebrates are smaller in size compared with vertebrates, gravity continues to have an effect on invertebrates biologically. Invertebrates share similar gravity-sensing systems found in mammals, differing depending on the context (e.g., terrestrial, aquatic, and aerial, described in further detail in reference 39). For example, on land, insects and crustaceans have joints with mechanosensitive hairs (e.g., hair plates) that adjust their joint angles to the direction of gravity. Invertebrates also have structures for measuring joint load, called campaniform sensilla in insects, lyriform organs in arachnids and centipedes, and cuticular stress detectors in crustaceans, sharing similarities to stretch and pressure sensors in human skin, deforming structurally and activating biochemical pathways in response to changes in gravity. Invertebrate muscles also contain stretch receptors (e.g., cerci, cone-shaped appendages extending horizontally from the rear of some crickets), highlighting different biological structures existing with invertebrates sensitive to gravity, though much remains to be discovered (Bender and Frye, 2009).

### 2.5 Plant adaptations to gravity

In addition to animals, plants also sense gravity, defining the direction of growth for their leaves and roots (e.g., gravitropism), and producing biological factors in response to gravity. For example, plants produce a hormone called auxin that guides the direction of plant structures’ growth. More specifically, auxin interacts with indole-3-acetic acid (IAA), with IAA inactivating the receptor auxin response factor (ARF) when auxin levels are low (e.g., lack of gravity). High auxin levels result in formation of the transcriptional repressor, Aux/IAA, and the Auxin signaling F-box protein co-repressor/auxin receptor, TIR/AFBs, which then degrades Aux/IAA and releases ARF to modulate auxin related genes (Lavy and Estelle, 2016). The auxin biochemical pathway (described in greater detail in ref. Sato et al., 2015; Su et al., 2017; Takahashi et al., 2021) is part of the Cholodny-Went theory, which describes differential plant gravitropism from auxin redistribution across plant components. Plants in a gravitational environment accumulate auxin in the lower side *versus* the upper side of shoots and roots in a horizontal position, causing the upward bending of shoots and downward bending of roots. Involved with the auxin redistribution process is the auxin carrier protein, PIN-FORMED (PIN); one example PIN protein is CsPIN1. Spaceflight experiments of cucumber seedlings on the Space Shuttle and the International Space Station have shown endodermal cells redistributing CsPIN1 while in space to laterally transport auxin from the upper to lower region of the seeds. While we have a working model for how plants sense gravity, e.g., Cholodny-Went theory, and have identified some relevant pathways (e.g., auxin), knowledge gaps continue to exist. Future areas of research should identify how the Cholodny-Went model is applicable to different plant types, as well as uncover more of the biological processes involved at the different layers of plant physiology (e.g., genetic to functional).

## 3 Additional effects of the space environment: Radiation

In addition to the effects of gravity on Earth and a lack of gravity while in space on biology, there are additional environmental effects from space travel including radiation exposure, a significant risk for future deep space missions to the Moon and beyond. The space environment includes galactic cosmic rays and solar particles as sources of particle radiation, composed of electrons and positrons (2%), protons (85%), helium nuclei (12%), and heavier ions referred to as high-energy and high-charge particles (HZE, 1%). Shielding can minimize crew exposure to space radiation, though the health impacts of these particles are still of concern due to their ionizing capabilities and energy deposition. Indeed on the ISS, astronauts are partially protected by the magnetic field of the Earth and shielding on station, but remain exposed to an average dose of 100–200 milliSievert (mSv) radiation per year (Cucinotta and Durante, 2006). On a mission to Mars, astronauts are predicted to be exposed up to 350 mSv per year for a ∼3-year mission (Zeitlin et al., 2013; Iosim et al., 2019).

In turn, there have been studies exploring the combined effects of radiation and microgravity exposure. These biological adaptations include increased oxidative stress, DNA damage, mitochondrial dysregulation, increased risk of cancer development, etc. (Cucinotta and Durante, 2006; Zeitlin et al., 2013; Iosim et al., 2019). Knowledge gaps still exist, in particular delineating spaceflight adaptations from a lack of gravity *versus* radiation, elucidating synergistic effects, differences between radiation zones (e.g., low Earth orbit *versus* cis-Lunar), as well as long-term effects from space radiation exposure.

## 4 Conclusion

Here are described in brief the varying roles gravity has with respect to biology in a few example contexts. These include different biological categories (e.g., animals, plants, etc.), different scales (e.g., cell to tissue to organ, etc.), and different mechanisms (e.g., genetic, protein pathways, structural, etc.). The role of and a lack of gravity is described as it relates with biological structure, function, and adaptation in various ways.

However, we have knowledge gaps that future research should aim to address. These include additional investigations studying gravity and a lack of gravity’s effects on microbes, plants, animals, humans, etc., evaluating the physiological adaptations of different systems (e.g., organs, tissues, cell types, organelles, macro- and micro-molecules, fluids, etc.), gender differences, and reproduction, development, and evolutionary mechanisms. Specific research questions for microbes include changes in biofilm and microbial community development, altered virulence, symbiosis, etc. Research questions for plants include changes in growth, development, metabolism, sense and reaction (e.g., further development of the Cholodny-Went theory), life cycles, etc. Research questions for animals include changes with sense and reaction, physiological structure and function, etc. Moreover, the thermal environment also has a significant effect on biology, and thermal exchange (e.g., convection) will be influenced by a lack of gravity; thus, thermal exchange is another significant factor for consideration of gravity’s effects on biology as well as for future research. Additional topical and research areas are described in greater detail in the following reviews (Elsaesser et al., 2023; Maarten, 2023).

Further research of the different layers of biochemical processes (e.g., gene, transcription, translation, intra- and inter-cellular signaling, structure, function, etc.) across organisms in response to gravity changes to identify shared pathways would advance the field of gravitational biology research as well. Moreover, the design and development of data-driven computational models that enable simulating and extrapolating across conditions would enable us to further the theoretical framework of models describing gravity’s effects on biology, as well as identify gaps that require experimental data that future projects can aim to address. Future areas of research should also include the combined effects of gravity with radiation, in preparation for future missions to the Moon and beyond as part of our Artemis program.

## Data Availability

The original contributions presented in the study are included in the article/supplementary material, further inquiries can be directed to the corresponding author.
